# Soya, maize and sorghum ready-to-use therapeutic foods are more effective in correcting anaemia and iron deficiency than the standard ready-to-use therapeutic food: randomized controlled trial

**DOI:** 10.1186/s12889-019-7170-x

**Published:** 2019-06-24

**Authors:** Peter Akomo, Paluku Bahwere, Hitoshi Murakami, Chrissy Banda, Elizabeth Maganga, Sylvester Kathumba, Kate Sadler, Steve Collins

**Affiliations:** 1 Valid Nutrition, Cuibín Farm, Derry Duff, Bantry, Co., Cork, Republic of Ireland; 2 Valid International, 35 Leopold Street, Oxford, OX4 1TW UK; 3 Nutrition Improvement Department, Ajinomoto Co., Inc., Tokyo, Japan; 4 MOH-Malawi, Lilongwe, Malawi; 5 Centre de Recherche en Epidémiologie, Biostatistique et Recherche Clinique, Ecole de santé publique, Université Libre de Bruxelles, Bruxelles, Belgium

**Keywords:** Anaemia, Iron deficiency, Severe acute malnutrition, Ready-to-use therapeutic food, Iron, Milk

## Abstract

**Background:**

The prevalence of anaemia and iron deficiency (ID) among children with severe acute malnutrition (SAM) and their correction during nutritional rehabilitation are not well documented. This study assessed anaemia and ID prevalence and their predictors at start of SAM treatment, and the efficacy of their treatment and effect on gut health of two novel Ready-To-Use Therapeutic foods (RUTF) prepared from soybean, maize and sorghum (SMS) with (MSMS-RUTF) or without added milk (FSMS-RUTF) compared to those of the standard formulation prepared from peanut and milk (PM-RUTF).

**Methods:**

This was a 3-arms parallel groups, simple randomised, controlled non-inferiority trial in 6–59 months old Central Malawian children with SAM. Anaemia was defined using altitude- and ethnicity-adjusted haemoglobin. Iron status was defined using soluble transferrin receptor (sT*f*R) and body iron stores (BIS). We used Pearson’s chi-square test, t-test for paired or unpaired data, Kruskal-Wallis test for between-arm differences as appropriate and logistic regression to identify independent predictors of anaemia or iron deficiency anaemia (IDA).

**Results:**

The sample size was 389. At admission, the prevalence [%(95%CI)] of anaemia was 48.9(41.4–56.5)% while that of ID and IDA were 55.7(48.6–62.5)% and 34.3(28.2–41.0)% when using sT*f*R criterion and 29.1(24.4–34.4)% and 28.9(23.7–34.9)% when using BIS criterion, respectively. At discharge, nutrition rehabilitation with SMS-RUTF was associated with the lowest prevalence of anaemia [12.0(6.9–20.3)% for FSMS-RUTF, 18.2(11.9–26.8)% for MSMS-RUTF and 24.5(15.8–35.9)% for PM-RUTF; *p* = 0.023] and IDA [7.9(3.4–17.3)% for FSMS-RUTF, 10.9(4.8–22.6)% for MSMS-RUTF and 20.5(10.7–35.5)% for PM-RUTF; *p* = 0.028]. SMS-RUTF was also associated with the highest increase in BIS [Change in BIS (95%CI)] among the iron deplete at admission [6.2 (3.7; 8.6), 3.2 (0.8; 5.6), 2.2 (0.2; 4.3) for the same study arms; Anova *p* = 0.045]. Compared to P-RUTF, FSMS-RUTF had the highest adjusted recovery rate [OR (95%CI = 0.3 (0.2–0.5) with *p* < 0.001 for FSMS-RUTF and 0.6 (0.3–1.0) with *p* = 0.068 for MSMS-RUTF]. No effect of iron content on risk of iron overload or gut inflammation was observed.

**Conclusions:**

Anaemia and ID are common among children with SAM. FSMS-RUTF is more efficacious in treating anaemia and correcting BIS among this group than PM-RUTF.

**Trial registration:**

This study was registered on 15 April 2015 (PACTR201505001101224).

**Electronic supplementary material:**

The online version of this article (10.1186/s12889-019-7170-x) contains supplementary material, which is available to authorized users.

## Background

Globally, severe acute malnutrition (SAM) affects about 16.4 million children aged 6–59 months, out of whom 23% currently access treatment using ready to use therapeutic food (RUTF) [1, 2]. Moreover, in Community-based Management of Acute Malnutrition (CMAM), RUTF is the recommended diet for children in this age group being treated for SAM [3]. RUTF is a fortified energy dense food intended to cover all nutritional needs during recovery from SAM [3–5]. The most common formulation of RUTF is based on peanut and milk. However, research has been ongoing to develop alternative formulations that are equally effective and are cheaper and more acceptable in settings where peanut is not popular.

SAM is known to be a pluri-deficiency disease combining macronutrients and multiple micronutrients [6]. Thus, diets for SAM treatment should aim at correcting micronutrients deficiencies in addition to fulfilling requirements for catch up growth [6–9]. Anaemia is one of the most widespread nutritional deficiencies affecting over 1.6 billion people worldwide [10]. The burden varies from one study to another but the most recent representative estimates indicate that overall, 47.4% children below 5 years are affected worldwide with the highest prevalence of 64.6% found among African children [10–13].

In children, anaemia (defined as a Haemoglobin below 110 g/L) is a clinical condition that results in weakness, difficulty in concentrating, susceptibility to infections, delayed cognitive and motor development and increased mortality [14, 15]. Anaemia can be classified into nutritional and non-nutritional anaemia, and is caused by several different factors including nutritional deficiencies, infections and inherited red blood cell disorders [16, 17]. Studies indicate that at least 50% of anaemia in children below 5 years of age from low and middle income countries is due to Iron deficiency (ID) [13, 14]. This has led to various initiatives including supplementation and food fortification to increase intake of iron [18, 19].

Studies conducted in Africa and Asia have reported prevalence of over 80% of anaemia among SAM children admitted in tertiary care hospitals [20–23]. To our knowledge, no study has reported the prevalence among children admitted into Community-based Management of Acute Malnutrition (CMAM) programmes, a treatment approach that allows early initiation of nutrition rehabilitation [24, 25]. The contribution of ID in anaemia during SAM is not well established, and available data suggest that the prevalence of ID may be context specific and depends on the co-existence of other factors contributing to the severity of SAM [26–29]. Despite the dearth of data on prevalence of IDA in SAM, the high prevalence of anaemia in children aged below 5 years and its serious clinical impacts underscore the importance of correcting ID during treatment of SAM. Moreover, the high iron demand during catch-up growth makes recovering SAM cases particularly susceptible to ID. The presence of ID means that these children are at low risk of iron supplementation side effects even in malaria endemic areas as most side effects are observed in iron replete individuals [30, 31]. Indeed, the World Health Organisation still recommends routine iron supplementation for prevention and treatment of IDA in malaria endemic areas where prevalence of IDA is also usually high [31].

Absorption of iron is influenced by both dietary and physiological factors, with the former being determined mainly by the form of iron and by the presence of inhibitors and enhancers in a particular meal [32]. Among other factors, phytic acid, milk proteins and calcium, have been identified as nutritional factors inhibiting iron absorption, while ascorbic acid enhances its absorption [32–34]. These factors are present in varying amounts in formulations based on soybean, maize and sorghum [34], and their effects on anaemia and iron status are not well understood.

The currently recommended iron content of RUTF of 10–14 mg/100 g tries to balance the need to correct ID and provide iron to support the rapid tissue synthesis required for catch-up growth with the need to minimise the production of free radicals and the risk of enhancing bacterial growth and virulence [3, 35, 36]. However, a study has shown that RUTF providing 22 mg per day of iron is less effective in maintaining or improving haemoglobin level than the therapeutic milk F100 providing 60 mg/day of iron [37]. More recently, we published results indicating that an RUTF containing 43.8 mg/100 g iron is more effective in treating anaemia than an RUTF containing 10-14 mg/100 g, however, the study was underpowered to demonstrate statistical difference in haemoglobin change in unadjusted analysis [38].

Concerns remain regarding the level of iron in therapeutic or supplementary food despite some studies showing the positive effect of iron supplementation on growth. These concerns are related to the potential side effects that some studies have attributed to iron fortified food of inducing an increase in the population of pathogenic bacteria in the gut that may induce intestinal inflammation [36]. Despite that a study conducted in South Africa showed that the iron form used in this study was not associated with increased bacterial growth, data comparing food with different iron contents are scarce making this a priority research area [36, 39].

This study aimed to determine the burden of anaemia among children with uncomplicated SAM and assess the efficacy of two novel RUTF formulations in correcting anaemia and ID during SAM treatment. The specific objectives were:

1. To determine the prevalence and risk factors of anaemia and ID among children with uncomplicated SAM,
2. To analyse the impact of the novel recipes with reduced milk content on the efficacy of treating anaemia and ID, and
3. To examine the impact of increased iron intake on intestinal inflammation.

## Methods

This study was embedded within a larger 3-arms parallel group, simple randomised, controlled trial assessing the efficacy in treating SAM of two novel formulations of RUTF, using the standard peanut-milk RUTF as control. The methodological aspects were reported in detail previously in the paper presenting on the main findings of the randomised clinical trial in recovery rates from SAM [40]. Below we only include the aspects of the methods required to understand the findings of this paper.

### Study design

This was a randomised clinical trial assessing the efficacy of 3 different formulation of RUTF in correcting anaemia and ID during the treatment of SAM. A sub-sample from the study population of the larger study, was randomly selected. The two principal outcomes of interest for this embedded study were: 1) prevalence and predictors of anaemia and ID at admission and at discharge; and 2) between arms difference in prevalence of anaemia and ID at discharge.

### Study setting and participants

The study was conducted at 21 day-care feeding centres scattered in three districts in Central Malawi (Lilongwe, Dedza and Mchinji). The altitude of this area ranges from 578 m to 1300 m above sea level. The incidences of malaria, intestinal parasitic infestation, viral and bacterial infections are high during childhood [41, 42].

A subsample of 389 children aged 6–59 months diagnosed with SAM (mid upper arm circumference [MUAC] < 115 mm or bilateral pitting oedema of any degree) who also had good appetite and no medical complication, were selected from the larger study sample of 1347 children randomly allocated in the 3 treatments group by the simple randomization (ratio 1:1:1) based on a computer generated random list and using a closed envelope method as already described [40]. The sub-sample was selected using a predetermined schedule, where the phlebotomist nurse visited 3 to 4 sites per day and collected samples for baseline data in all those who were admitted 48 h before the visit. The collection of samples for baseline data was conducted across all the sites and was stopped when the required sample was reached. These same subjects were followed for collection of further samples at the subsequent sampling time-points.

### RUTF formulations

The two new RUTF formulations were based on extruded soya, maize and sorghum, one containing no milk (FSMS-RUTF) and the other 9% milk (MSMS-RUTF). The control RUTF (PM-RUTF) was based on peanuts and dried skim milk and contained 28% milk. As shown in Table 1, all the RUTFs were designed to meet the World Health Organisation (WHO) recommendations for RUTF mineral and vitamin levels [1] with the exception of iron and zinc. Iron, zinc and vitamin C levels in FSMS-RUTF and MSMS-RUTF were increased to attain a phytic acid / iron molar ratio, ascorbic acid / iron weight ratio and zinc / iron weight ratio of < 2.5, 3.0–16.0 and 0.8–3.5, respectively to enhance iron and zinc absorption [43, 44]. Iron was in the form of ferrous sulphate monohydrate. All samples were identical in packaging except a colour code which was not understood by the participants and field implementers.

**Table 1:** Nutritional composition of the study foods

		Value / 100 g		
Component	Unit	FSMS-RUTF^a^	MSMS-RUTF^b^	PM-RUTF^c^
Energy	kcal	532	544	545
Protein	g	18.4	16.6	15.6
Fat	g	34.2	36.0	33.8
Fibre	g	7.1	4.8	1.9
Calcium	mg	571	399	434
Phosphorus	mg	503	493	351
Iron	mg	35.1	31.6	10.5
Zinc	mg	19.5	19.9	11.1
Selenium	μg	26	25	27
Vitamin A	mg RE^d^	1.25	1.16	1.18
Thiamine	mg	1.28	1.12	0.97
Riboflavin	mg	1.63	1.97	3.20
Vitamin C	mg	323	306	87
Vitamin B6	mg	0.99	0.93	0.66
Vitamin B12	μg	2.5	2.6	3.2
Folate	μg	210	200	268
Niacin	mg	7.54	7.94	7.6
Pantothenic acid	mg	5.36	4.73	4.5
Biotin	μg	86	81	80
Choline	mg	90	70	–
Phytic acid	g	0.465	0.333	0.251
Phytic acid/Iron molar ratio	–	1.12	0.89	2.02
Phytic acid/zinc molar ratio	–	2.36	1.66	2.24
Ascorbic acid/Iron molar ratio	–	2.93	3.08	2.64
Ascorbic acid/Iron weight ratio	–	9.20	9.68	8.29
Calcium/phosphorus weight ratio	–	1.14	0.81	1.24
Zinc/copper weight ratio	–	13.18	13.27	6.94
Zinc/iron weight ratio	–	0.56	0.63	1.06

^a^*FSMS-RUTF* Milk Free Soya-Maize-Sorghum based Ready-To-Use Therapeutic Food

^b^*MSMS-RUTF* Milk Soya-Maize-Sorghum based Ready-To-Use Therapeutic Food

^c^*PM-RUTF* Peanut paste based Ready-To-Use Therapeutic Food

^d^*RE* Retinol equivalent

### Treatment protocol and Iron intake

The nutrition and medical management of children in all study groups were similar and followed the Malawi national guidelines for the management of acute malnutrition with the exception of the day-care approach and the fact that children were allowed to eat the RUTF ad libitum. Study assistant nurses fed the children with the support of the caregivers and closely monitored intake and occurrence of symptoms and clinical signs such as appetite, diarrhoea, vomiting, abdominal pain, flatulence, fever, skin eruption, cough and respiratory distress. No iron was added to the treatment in case of mild or moderate anaemia. As per the national protocol, a blood transfusion was needed only if the haemoglobin was < 5 g/dl.

### Data collection and procedures

Nutritional and morbidity parameters were monitored and recorded daily on specially designed forms. Blood samples for measurement of anaemia (haemoglobin, haematocrit and full blood count), iron status (ferritin, soluble transferrin receptor), retinol-binding protein and inflammation (C-reactive protein, and alpha-1-acid glycoprotein) parameters were measured on admission and at discharge. Trained paediatric phlebotomists collected blood by antecubital or metacarpal venipuncture into appropriate tubes provided by the Lilongwe University of North Carolina research laboratory. All the blood specimens were immediately stored in CubeCooler™ and kept at 4 °C [45] and delivered to the Lilongwe research laboratory within 6 h. Samples for the measurement of haemoglobin, haematocrit and full blood count were analysed at the Lilongwe UNC laboratory immediately after delivery. For other parameters, samples were later shipped to Germany for the measurement of the 5 plasma proteins [ferritin, soluble transferrin receptor (sTfR), retinol-binding protein (RBP), C-reactive protein, and alpha-1-acid glycoprotein (AGP)]. At the Lilongwee laboratory, cells and plasma were separated within 24 h and the plasma was subsequently stored at − 80 °C until shipping to Germany. Before shipping to Germany, the samples were unfrozen to allow for the transfer of 0.2 ml of plasma into the VitMIN laboratory special pre-labelled storage tubes (Willstaett, Germany). All the samples were refrozen after the transfer. During shipping to Germany, the sample cold-chain was maintained using cooler boxes with dry ice.

A combined sandwich ELISA method was used to analyse 5 plasma proteins [ferritin, soluble transferrin receptor (sTfR), retinol-binding protein (RBP), C-reactive protein, and alpha-1-acid glycoprotein (AGP)] for determination of iron status [46].

To assess the impact of the different study RUTFs on gut inflammation, stools samples were collected from the subset of subjects from whom fresh stools could be obtained during the day of the visit by the samples collectors in plastic pots at baseline, at 3 weeks and at discharge for the measurement of calprotectin [47, 48]. A quantitative point-of–care chromatographic immunoassay kit (Quantum Blue®, Alpha laboratories, Hampshire, UK) was used for the measurement [49, 50]. As per the manufacturer’s instructions, two drops of homogenised stool were applied inside a plastic cassette and the cassette inserted into a portable electronic reader that displayed numerical value representing calprotectin concentration. The cassette reader was calibrated for each day of tests.

### Sample size

Sample size was calculated to demonstrate the non-inferiority of the new RUTFs in terms of haemoglobin change [40]. A total of 192 children studied at both admission and discharge were required to be 90% sure that the lower limit of a one-sided 95% confidence interval would be above the non-inferiority limit of − 0.5 g dl-1 indicating no difference between the arms. We increased this sample to a minimum of 300 children at admission and 225 (75 per arm) at discharge, to account for loss to follow up during nutrition rehabilitation due to defaulting, death, withdrawal of consent for blood collection, difficulties in blood collection or the requirement for a transfusion during treatment. This sample size was also sufficient for the determination of the prevalence of anaemia and ID at admission (calculated sample size = 292 children assuming prevalence of 50 ± 5%) and discharge (calculated sample size of 233 children assuming prevalence of 25 ± 5%), the detection of a trend in the prevalence of anaemia at discharge equivalent to a 9% difference between groups and the existence of a difference in intestinal inflammation between the study arms.

### Variable transformation, adjustments and definitions

Individual haemoglobin values were adjusted for altitude and ethnicity according to the methods by WHO [51] and Sullivan et al. [52]. The adjusted values were then used to define anaemia using the recommended cut-off for children aged 6–59 months of 110 g/L [51, 52].

As β-thalassemia trait is common among Malawian children, we calculated the Mentzer index to serve as a proxy of presence of β-thalassemia trait [16, 53, 54]. The index is calculated as the quotient of the mean corpuscular volume (MCV, in fL) divided by the red blood cell count (RBC, in Millions per microliter). β-thalassemia trait was assumed if the index was < 13 [53, 54].

Plasma ferritin, soluble transferrin receptor (sT*f*R) and body iron stores (BIS) were used to describe iron status [55, 56]. As these biomarkers are impacted by inflammation, they were adjusted using the inflammation biomarkers C-reactive protein and alpha-1-acid glycoprotein (AGP), according to the method proposed by Thurnham et al. [57]. We report iron sufficiency or deficiency using BIS (ratio of inflammation-adjusted Plasma Ferritin to inflammation-adjusted sT*f*R [58] as a continuous variable with a BIS < 0 mg.kg^− 1^ indicating ID, a BIS between 0 and 2.9 mg.kg^− 1^ low iron status and a BIS ≥3 mg.kg^− 1^ indicating normal iron status. With adjusted sTfR, a value > 8.3 mg/L indicated ID while a value below this cut-off indicated absence of ID. We used Plasma Ferritin to define excess body iron stores or iron overload [59], taking a cut-off > 100 μ/L to define excess body iron stores [59].

### Data management and analysis

Data were double entered by two enumerators into a customized Epidata database prepared for this study [60]. Quality of data entry was monitored by the supervisors who cross-checked a random selection of 10% of the records. Cleaned data were exported to stata-13 for analysis [61].

Some data for key biological markers of anaemia and iron status at discharge data collection point could not be obtained. An assessment of the reasons identified the following: discontinuation of collection of specimens for this sub-study when the number that the budget allowed to be included was reached; child dropout from the study; child death; and no return after being transferred for management of complication. The budget shortage was due to the unexpected rise in prices of the tests obliging us to reduce the sample of participants to be surveyed at discharge. The retrospective follow up of defaulters revealed that the defaults were attributed to relocation, death or mothers related reasons (fatigue due to advanced pregnancy, delivery of new baby, taking care of a hospitalised household member, divorce and farming obligations). As all these reasons are responsible of missing values that can be classified as “missing completely at random” we opted for complete case analysis approach that drops the missing values in the analysis [62, 63].

Means and standard deviations (SD), medians and interquartile ranges (IQR) or proportions and 95% Confidence intervals (95%CI) were used to describe the admission and discharge parameters, as appropriate. All 95% CIs used clustered robust estimates of the variance to account for clustering at the level of the day care centre. Means were compared using t-test for paired or unpaired data, as appropriate, medians were compared using the Kruskal-Wallis test and proportions compared using the Pearson’s chi-square test or the chi-square test for trend as appropriate. We used logistic regression to identify predictors of anaemia and IDA at admission and discharge. The predetermined candidate predictors were study arm, age, sex, MUAC of admission, presence of oedema at admission, adjusted haemoglobin of admission, length of stay in programme, daily RUTF intake and presence of β-thalassemia trait. The final model was obtained using the approach recommended by Greenland for the selection of variables [56]. We started with the full model with all the potential predictors listed above and thereafter manually removed non-significant variables one by one using the *p*-value and the change-in-estimate method [64]. Multivariable linear regression was used to obtain adjusted prevalence at discharge of anaemia, ID and IDA. The adjustment variables were gender, mother education level, anaemia at admission, iron status of admission as measured by BIS, and inflammation category of admission.

## Results

Out of the 1303 children screened and accepted into the larger efficacy study, 389 were randomly selected for this embedded study of whom haemoglobin and iron status at admission was determined in 386 and 343, respectively. The other 46 were excluded due to difficulties of obtaining adequate blood samples (*n* = 43) and withdrawal of consent (*n* = 3). Recruitment and follow up for this study was conducted between September 2015 and June 2016. Table 2 presents the characteristics at admission of the enrolled children. The sample was balanced in terms of sex and breastfeeding status. There were more children below 24 months and the great majority had both parents alive with almost all the heads of the households being subsistence farmers. The three groups were different with regard to maternal education status, with PM-RUTF group having the lowest proportion of mothers with no education. Overall, less than 20% of the mothers reached the secondary level of education. There were marginally insignificant differences in gender and inflammation among the three groups. Access to safe drinking water was high but utilisation of insecticide treated net (ITN) was reported by less than 50%. The two clinical forms of SAM were equally represented in the sample. The majority had some form of inflammation with elevated values of C-reactive protein or AGP. The Mentzer index classified only few of the children as probably having β-thalassemia trait. The between arms comparison showed that there was no difference in the other parameters evaluated.

**Table 2:** Characteristics of study subjects at admission according to study arms^a^

Parameter	TOTAL *n* (%)	FSMS-RUTF *n* (%)	MSMS-RUTF *n* (%)	PM-RUTF *n* (%)	*p*-value^*^
Sex					
Male	202 (51.5)	76 (56.7)	47 (43.1)	79 (53.0)	0.097
Female	190 (48.5)	58 (43.3)	62 (56.7)	70 (47.0)	
Age					
< 24mo	227 (57.9)	71 (53.0)	67 (61.5)	89 (59.7)	0.350
> 24mo	165 (42.1)	63 (47.0)	42 (38.5)	60 (40.3)	
Breastfeeding status					
Breastfed	188 (48.1)	59 (55.6)	55 (50.5)	74 (49.7)	0.567
Not breastfed	203 (51.9)	74 (44.4)	54 (49.5)	75 (50.3)	
Highest level of education of mother					
None	163 (41.6)	56 (41.8)	34 (31.2)	73 (49.0)	0.040
Primary	153 (39.0)	52 (38.8)	54 (49.5)	47 (31.5)	
Secondary or higher	76 (19.4)	26 (19.4)	21 (19.3)	29 (19.5)	
Principal activity of head of the household					
Subsistence farming	368 (93.9)	126 (94.0)	103 (94.5)	139 (93.3)	0.920
Others	24 (6.1)	8 (6.0)	6 (5.5)	10 (6.7)	
Father vital status					
Dead	4 (1.0)	1 (0.7)	0 (0.0)	3 (2.0)	0.262
Alive	388 (99.0)	133 (99.3)	109 (100)	146 (98.0)	
Mother vital status					
Dead	7 (1.8)	3 (2.8)	0 (0.0)	4 (2.7)	0.244
Alive	385 (98.2)	131 (97.8)	109 (100)	145 (97.3)	
Drinking water source					
Improved	297 (76.6)	101 (75.9)	81 (75.0)	115 (78.2)	0.817
Unimproved	91 (23.4)	32 (24.1)	27 (25.0)	32 (21.8)	
Use of insecticide treated net					
No insecticide treated net used	173 (44.6)	52 (39.4)	50 (46.3)	71 (48.0)	0.324
Using insecticide treated net	215 (55.4)	80 (60.6)	58 (53.7)	77 (52.0)	
Inflammation status					
No inflammation	41 (11.7)	13 (11.0)	15 (15.3)	13 (9.7)	
Incubation	16 (4.6)	5 (4.2)	8 (8.2)	3 (2.2)	
early convalescence	178 (50.9)	59 (50.0)	53 (54.1)	66 (49.3)	0.086
late convalescence	115 (32.8)	41 (34.8)	22 (22.4)	52 (38.8)	
Mentzer index^b^					
< 13: beta-thalassemia likely	60 (15.3)	19 (14.3)	20 (18.4)	21 (14.1)	0.591
≥13: beta-thalassemia unlikely	331 (84.7)	114 (85.7)	89 (81.6)	128 (85.9)	
Clinical form of SAM^c^ at admission					
Oedematous	217 (55.4)	72 (53.7)	64 (58.7)	81 (54.4)	0.704
Non-oedematous	175 (44.6)	62 (46.3)	45 (41.3)	68 (45.6)	

^*^p = value of chi-square test comparing the 3 arms

^a^Study arms: *FSMS-RUTF* Milk Free Soya-Maize-Sorghum based Ready-To-Use Therapeutic Food, *MSMS-RUTF* Milk Soya-Maize-Sorghum based Ready-To-Use Therapeutic Food and *PM-RUTF* Peanut milk based Ready-To-Use Therapeutic Food

^b^*Mentzer index* mean corpuscular volume/red blood cell count

^c^*SAM* severe acute malnutrition

### Prevalence of anaemia, iron deficiency and iron deficiency anaemia at admission

Out of the 386 children for whom haemoglobin was measured on admission (3 children withdrew consent after randomisation), anaemia was diagnosed in [% (95%CI)] 48.9(41.4–56.5)%. Table 3 presents the distribution of anaemia per selected parameters. Children with β-thalassemia trait had low prevalence of anaemia when compared to those without the trait while those with inflammation at incubation and early convalescence had increased risk of anaemia when compared to those with no inflammation. Logistic regression analysis (detailed table provided as Additional file 1) confirmed the protective effect for anaemia of having β-thalassemia trait [Adjusted Odds Ratio (AOR) with absence of the trait as reference: (0.4(0.2–0.9); *p* = 0.036] and high likelihood of anaemia in presence of inflammation at incubation phase [AOR (5.1(1.6–16.8); *p* = 0.007] or at early convalescence phase [AOR (6.5(2.6–16.4); *p* < 0.001]. The other predictors of anaemia were age (in months) at admission [AOR (0.94(0.90–0.97); *p* = 0.001] and weight (in kg) at admission [AOR (1.5(1.1–2.0); *p* = 0.016].

**Table 3:** Prevalence of anaemia, iron deficiency and iron deficiency anaemia at admission

Criteria	n	%	(95%CI	
*Prevalence of anaemia*				
All children	386	48.9	(41.4–56.5)	
Anaemia by admission age				
< 24 months	226	50.3	(43.3–56.7)	
≥ 24 months	160	46.9	(39.7–57.3)	
Anaemia by sex				
Boys	200	44.5	(34.2–55.2)	
Girls	186	53.7	(45.0–62.2)	
*Prevalence of iron deficiency*				
By plasma sTfR (> 8.3 mg/L)				
All children	343	55.7	(48.6–62.5)	
No β-thalassemia	290	53.4	(45.0–61.7)	
No inflammation	41	64.9	(44.4–81.0)	
No inflammation and β-thalassemia	29	58.6	(36.5–77.7)	
Children < 24 months still breastfed	154	70.8	(62.9–77.6)	
Children< 24 months not breastfed	46	45.6	(32.2–59.7)	
Among non-anaemic	177	42.9	(35.5–50.6)	
Among anaemic children	166	69.3	(61.7–76.2)	
By body iron stores criterion				
Low iron stores (0 and 2.9 mg.kg^−1^)	343	29.1	(24.4–34.4)	
Depleted iron stores (< 0 mg.kg^−1^)	343	26.2	(21.8–31.1)	
Low or depleted iron stores(< 3 mg.kg^−1^)				
Among all children	343	55.4	(50.0–60.7)	
Among non-anaemic children	177	52.0	(44.3–59.5)	
Among anaemic children	166	59.0	(51.1–66.6)	
*Prevalence of iron deficiency anaemia*				
By plasma sTfR criterion(> 8.3 mg/L)	343	33.5	(28.5–38.8)	
By body iron stores criterion (< 3 mg/kg)	343	28.6	(23.8–33.7)	

Table 3 also presents observed ID prevalence. The prevalence is higher using sT*f*r criterion [% (95%CI)] [55.7(48.6–62.5)%] than with BIS criterion [29.1(24.4–34.4)%] but similar to that observed when the categories low BIS and depleted BIS are combined [55.4(50.0–60.7)%]. Among children below 24 months of age, those still breastfed had a significantly higher prevalence of ID (*p* = 0.004). A third of all enrolled children had IDA and > 60% of the anaemia was associated with ID (Table 3). ID was also observed in non-anaemic children (Table 3). The independent predictors of IDA were largely similar to those for anaemia (Additional files 1 and 2); they included the absence of β-thalassemia trait (figures for IDA) [OR (0.4(0.2–0.9); *p* = 0.036], presence of inflammation at incubation phase [OR (5.1(1.6–16.8); *p* = 0.007] and at early convalescence phase [OR (6.5(2.6–16.4); *p* < 0.001], age (in months) at admission [OR (0.9(0.9–1.0); *p* = 0.001], weight (in kg) at admission [OR (1.5(1.1–2.0); *p* = 0.016] all predicted both anaemia and IDA. Sex, utilisation of ITN, MUAC at admission and nutritional oedema status were not predictors of IDA (Additional file 2).

### Effect of RUTF used for nutritional rehabilitation on anaemia and iron deficiency

In this study, iron intake was [mean (IQR)] 96.9 (71.0–132.4), 84.3 (58.1–104.7), and 28.0 (22.2–37.7) mg d^− 1^ in children aged 6–23 mo at admission and 113.0 (87.2–151.8), 101.8 (78.5–136.6), and 42.5 (34.8–51.2) mg d^− 1^ in children aged 24–59 mo at admission for the FSMS-RUTF, MSMS-RUTF, and PM-RUTF arms, respectively.

From the 386 surveyed at admission, 266 (68.9%) were assessed at discharge. The reasons for some not being assessed at discharge were reduction of the sample size because of budget constraint (*n* = 49), defaulting (*n* = 54), not returned from referral (*n* = 12) and death (*n* = 5). The proportion of missing data were 36.1(27.9–45.0) % for the FSMS-RUTF arm, 29.0(20.6–38.5) % for MSMS-RUTF arm and 28.8(21.7–36.8) % for PM-RUTF (*p* = 0.291). The distribution of reasons for missing data were not significantly different across study arms (data not shown).

Out of the 266 children assessed at discharge, 18.8 (13.4–25.7) % were anaemic. The figure was 17.5 (12.1–24.7) % for those discharged as cured (*n* = 257), 16.9 (11.2–24.6) % for those without β-thalassemia trait at admission and discharged as cured (*n* = 219), 6.5 (2.4–16.7) % for those without anaemia at admission (*n* = 107) and 26.2 (18.3–35.9) % for those who were anaemic at admission (*n* = 107). Table 4 presents the data disaggregated by type of RUTF used for nutrition rehabilitation. The results show a linear trend in the prevalence of anaemia related to the milk content of the RUTF with the prevalence lowest in the FSMS-RUTF and highest in the PM-RUTF. This trend was seen in both those with and without anaemia at admission. A similar trend was observed when comparing haemoglobin change between admission and discharge (Additional file 3). FSMS-RUTF was associated with greater increase of haemoglobin among children without the β-thalassemia trait [Δ (SD)] = 2.9 (1.5) for FSMS-RUTF (*n* = 35), 2.6 (2.0) for MSMS-RUTF (*n* = 36) and 2.0 (1.4) for PM-RUTF (*n* = 42); *p* = 0.0271]. In logistic regression analysis adjusting for haemoglobin at admission and average daily intake of RUTF (Additional file 4), both FSMS-RUTF [OR (95%CI = 0.3 (0.2–0.5); *p* < 0.001] and MSMS-RUTF [OR 0.6 (0.3–1.0); *p* = 0.068] were independently associated with reduced likelihood of being anaemic at discharge and there was a statistically significant linear trend associated with the milk content of the products (*p* < 0.001).

**Table 4:** Adjusted prevalence of anaemia, ID and IDA at discharge across study arms^a^

	n	%(95%CI)^f^	Unadjusted difference		Adjusted difference^b^		*p*-value^e^
			Diff^g^	(95%CI)	Diff	(95%CI)	
Prevalence of anaemia^c^ at discharge							
*All children*							
FSMS-RUTF	83	12.0 (6.9–20.3)	Base		Base		
MSMS-RUTF	77	18.2 (11.9–26.8)	6.1	(−0.6; 18.3)	7.6	(−3.9; 19.0)	0.195
PM-RUTF	106	24.5 (15.8–35.9)	12.5	(1.2; 23.7)	12.6	(2.1; 23.1)	0.019
*Without anaemia at admission*							
FSMS-RUTF	43	4.6 (1.0–19.2)	Base		Base		
MSMS-RUTF	35	5.7 (0.8–31.5)	1.1	(−9.1; 11.2)	5.7	(−5.2; 16.6)	0.303
PM-RUTF	59	11.9 (5.5–23.7)	7.2	(−3.6; 18.0)	7.1	(−2.4, 16.5)	
*With anaemia at admission*							
FSMS-RUTF	38	18.4 (10.0–31.3)	Base		Base		
MSMS-RUTF	40	30.0 (20.0–42.3)	11.6	(−8.8; 32.0)	12.8	(−0.3; 35.0)	0.254
PM-RUTF	45	40.0 (26.0–55.9)	21.6	(1.7; 41.4)	22.2	(2.9; 41.5)	0.024
Prevalence of iron deficiency^d^ at discharge							
*All children*							
FSMS-RUTF	64	42.2 (29.1–56,4)	Base		Base		
MSMS-RUTF	46	60.9 (49.4–71.2)	18.7	(−0.1; 37.5)	25.3	(6.6; 44.0)	0.008
PM-RUTF	84	58.3 (44.9–70.7)	16.1	(−0.1; 34.2)	20.6	(4.8;36.4)	0.011
*Iron deficient at admission*							
FSMS-RUTF	36	47.2 (29.5–65.7)	Base		Base		
MSMS-RUTF	21	85.7 (56.1–96.6)	38.5	(15.5; 61.2)	38.7	(17.7; 59.6)	< 0.001
PM-RUTF	43	83.7 (73.9–90.3)	36.5	(16.2; 56.7)	35.0	(14.7; 55.2)	0.001
Prevalence of iron deficiency anaemia at discharge							
All children							
FSMS-RUTF	63	7.9 (3.4–17.3)	Base		Base		
MSMS-RUTF	46	10.9 (4.8–22.6)	2.9	(−8.4; 14.3)	2.9	(−8.5; 14.4)	0.611
PM-RUTF	83	20.5 (10.7–35.5)	12.5	(1.4; 23.6)	10;8	(0.6; 21.1)	0.039

^a^Study arms: *FSMS-RUTF* Milk Free Soya-Maize-Sorghum Based Ready-To-Use Therapeutic Food, *MSMS-RUTF* Milk Soya-Maize-Sorghum Based Ready-To-Use Therapeutic Food and *PM-RUTF* Peanut milk based Ready-To-Use Therapeutic Food

^b^adjusted for age, gender, mother level of education, anaemia at admission and presence of iron deficiency using the body iron store based criterion

^c^Anaemia diagnosed based on altitude- and ethnicity-corrected haemoglobin

^d^Iron status according to inflammation-corrected soluble transferrin receptor values

^e^*p*-value for the adjusted difference obtained by multivariable linear regression

^f^*CI* confidence interval

^g^*Diff* difference

Regarding ID and IDA, Table 4 also shows that FSMS-RUTF arm had higher cure rates and lower frequency of new episodes for both ID and IDA than the two other arms. The linear trend was also statistically significant.

**Fig. 1:**
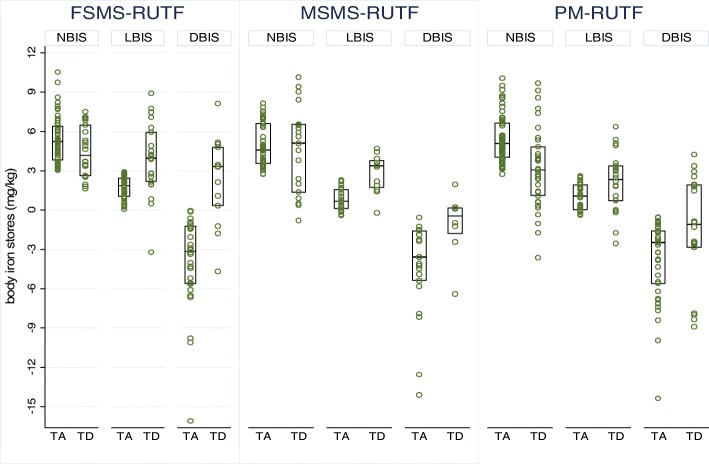
Body iron stores at admission (TA) and discharge (TD) according to study arm and body iron status category of admission. Study arms: FSMS-RUTF = Milk Free Soya-Maize-Sorghum Based Ready-To-Use Therapeutic Food, MSMS-RUTF = Milk Soya-Maize-Sorghum Based Ready-To-Use Therapeutic Food and PM-RUTF = Peanut milk based Ready-To-Use Therapeutic Food; Body iron stores categories: NBIS=Normal body iron stores/no iron deficiency, LBIS = Low body iron stores, DBIS = Depleted Iron stores; Assessment time: TA = at admission and TD = at discharge

Figure 1 shows the distribution of individuals’ values of BIS at admission and discharge according to iron status at admission. Overall, there was an increase in BIS but with variations according to type of RUTF used for nutrition rehabilitation and according to BIS category at admission (Additional file 5). Some children with normal BIS at admission became iron deficient during the course of nutrition recovery. For children with normal iron stores, the BIS decreased in all study arms by an average (Δ(95%CI) of − 0.9 (− 1.9; 0.1) for the FSMS-RUTF arm (*p* = 0.074), − 0.3 (− 1.9; 1.3) for the MSMS-RUTF arm (*p* = 0.686) and − 2.2 (− 3.3; − 1.1) for the PM-RUTF arm (*p* < 0.001). By contrast, children with BIS indicative of low iron status or iron deficiency at admission all showed significant increases in iron stores during treatment with observed changes (Δ(95%CI) of 2.2 (0.8; 3.5), 1.9 (0.9; 2.8) and 1.1 (0.1; 2.1) for FSMS-RUTF (*p* = 0.003), MSMS-RUTF (*p* = 0.001) and PM-RUTF (*p* = 0.033) respectively in the low iron status category and 6.2 (3.7; 8.6) for FSMS-RUTF group (*p* < 0.001), 3.2 (0.8; 5.6) for MSMS-RUTF (*p* = 0.011), 2.2 (0.2; 4.3) PM-RUTF (*p* = 0.034) in the iron depleted category. The frequency of children with the inflammation-adjusted Plasma Ferritin at discharge > 100 μg/L indicative of excess iron reserve were 1.6% (1/64) in the FSMS-RUTF arm, 4.3% (2/46) in the MSMS-RUTF arm and 4.8% (4/84) in the PM-RUTF arm (*p* = 0.559). For children with β-thalassemia trait, only one had adjusted Plasma Ferritin > 100 μg/L at discharge. This child was from the PM-RUTF arm.

### Effect of RUTF used for nutrition rehabilitation on gut inflammation

**Fig. 2:**
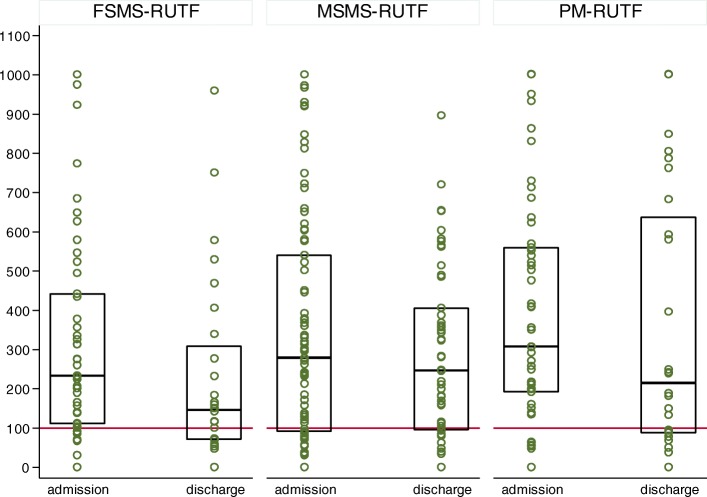
Gut inflammation at admission and discharge according to study arm. Study arms: FSMS-RUTF = Milk Free Soya-Maize-Sorghum Based Ready-To-Use Therapeutic Food, MSMS-RUTF = Milk Soya-Maize-Sorghum Based Ready-To-Use Therapeutic Food and PM-RUTF = Peanut milk based Ready-To-Use Therapeutic Food

Gut inflammation as measured by concentration of calprotectin in stools was high in all study arms at admission and remained high after 3 weeks of treatment and at discharge, with no statistically significant difference across the study arms. The median values of the calprotectin in stools at admission [median (interquartile range): 233.0(110.0–442.0) μg/g of stool for FSMS-RUTF, 278.0(91.0–540.0) μg/g of stools for MSMS-RUTF, 307.0(192.0–560.0) μg/g of stools for PM-RUTF; *p* = 0.385] and discharge [median (interquartile range): 146.0(69.5–308.0) μg/g of stools for FSMS-RUTF, 247.0(94.0–406.0) μg/g of stools for MSMS-RUTF, 213.5(87.5–637.5) μg/g of stools for PM-RUTF; *p* = 0.263] remained well above the cut-off for presence of gut inflammation of 100 μg/g of stool until discharge (Fig. 2).

## Discussion

In this study, we have shown that prevalence of anaemia, ID and IDA are high in Malawian children with uncomplicated SAM admitted into a CMAM programme, above the WHO criterion of 40% defining a severe public health problem and much higher than the 29% reported by the most recent community-based study among Malawian children below 5 years [16, 65]. Similar high prevalence of anaemia among children with SAM have been reported in other countries and continents [66, 67], indicating that SAM children should be considered as an “at risk group” for anaemia.

Emerging evidence suggests that anaemia in low income countries is multifactorial with dietary factors an important cause [20, 28, 29, 67]. Our results indicate that a high proportion of Malawian children who develop SAM during hunger seasons, which occur around November to March when family food reserves are largely depleted, have IDA and a pre-existing iron deficiency before developing SAM indicating that the usual factors causing iron deficiency are also in play for children with SAM [26, 68–70]. This finding contradicts the historical view that children with SAM usually have elevated body iron at diagnosis and supports the current practise of presumptive treatment of IDA during treatment of SAM.

This study also demonstrates that the content of cow’s milk in RUTF is inversely related to the product’s efficacy in treating anaemia and replenishing Body Iron Stores (BIS). This is more demonstrated by the difference in efficacy between the two SMS-based formulations which had similar phytic acid/iron and ascorbic acid / iron molar ratios and ascorbic acid / iron weight ratios. It is well known that casein, whey protein and calcium, that are all abundant in cow’s milk, inhibit iron absorption [15, 33, 71]. In this study, the milk-free FSMS-RUTF product containing 35.1 mg Fe / 100 g performed significantly better than MSMS-RUTF containing 9.3% cow’s milk and comparable iron content of 31.6 mg / 100 g. In turn, the MSMS-RUTF performed significantly better than the standard PM-RUTF product containing 28.2% cow’s milk and 10.5 mg Fe /100 g. These results are similar to those of a study conducted in Burkina Faso that also reported a better absorption of zinc when provided as tablet than when given concurrently with a low dose lipid-based nutrient supplement (LNS) containing cow’s milk powder [72]. In order to achieve optimal mineral delivery through ready to use foods, these findings suggest that further research is needed to determine the optimal balance between levels of mineral fortification and the composition of the food including the content of milk.

The superior performance of the pulse/grain-based formulations compared to the milk-based formulation demonstrates that the high content of phytates in cereals and pulses is not necessarily a barrier to using these ingredients in the manufacture of RUTFs. To address this problem, the iron and vitamin C levels in the two new RUTF formulations were increased to achieve phytic acid: iron molar ratio and vitamin C: iron weight ratios that are optimum for iron absorption [43, 44, 73].

A potential problem with increasing the level of iron is that unabsorbed iron could change the gut microbiome and promote the growth of pathogens [47, 74–76]. This is particularly relevant as this study was conducted in a malaria-endemic setting where some studies have suggested that iron supplementation may enhance morbidity in iron replete children [77] and controversy exists over whether foods in such settings should be fortified with iron. However as reported in our previous study, no differences in morbidity was observed in our studies between the high iron and low iron RUTF arms, indicating that the iron content of the foods was not associated with an increased frequency of episodes of fever [40]. This finding mirrors that of a previous study using an LNS with a much higher level of iron for a long period of 6 months [78].

The absence of any differences in stools calprotectin concentration, a marker of intestinal inflammation between the study arms at any of the three points of measurement also indicates that the higher levels of iron were not associated with increased gut mucosal inflammation [47, 79]. These findings again reflect those of a study conducted among South African children that concluded that supplementation with ferrous sulphate had no detrimental effect on microbiota [39]. However, it is interesting to note that high faecal calprotectin levels persisted in all three study arms, a finding reported elsewhere in children recovering from SAM [80] and suggestive of persistent intestinal inflammation that is not completely resolved by the treatment. Although several studies have shown that nutrition rehabilitation with RUTF is associated with improvement of the microbiota, the improvement is incomplete and transient [81, 82]. In children with cystic fibrosis who have similar gut mucosal abnormalities to those seen in SAM, the use of probiotics has been associated with improvements in mucosal structure [83, 84] suggesting that adding probiotics into RUTF may help to achieve sustained normalisation of intestinal mucosa. Interestingly, 8 years ago we published a pioneering study that assessed the effect of adding probiotic in RUTF and yielded promising results suggesting positive effect on mortality [85].

Increasing the iron was also not associated with iron overload and in this study changes in BIS were inversely proportional to BIS at the start of treatment, demonstrating that in the presence of tissue ID there is an up-regulation of intestinal absorption and vice versa. This indicates that tissue iron status in children with SAM still plays the central role in regulating (upregulating and downregulating) iron intestinal absorption and mobilisation from body stores [86–89]. As a result, during the usual duration of CMAM treatment biological regulation mechanisms appear able to keep tissue iron concentration below the excess cut-off and the risk of excess body iron stores is limited. This finding contradicts observations gathered before the advent of CMAM that reported a deregulation of iron metabolism at the time of admission into therapeutic feeding programmes [24, 86, 90]. In practice, it means that the fear of an increased absorption of iron during SAM is not a reason to prevent upward revision of the recommended iron density for RUTF used for uncomplicated SAM.

An important finding from this study is that none of the formulations used, even the higher iron non-milk formulation, adequately corrected ID and IDA by the time of reaching CMAM anthropometric discharge criteria in a substantial proportion of children. This is a serious deficiency. The improved performance of the plant-based RUTFs containing higher quantities of iron and the absence of negative side effects of increasing the iron content [38, 40] strongly suggest that the current recommendation on iron density of RUTF needs to be revised upwards [3].

We also found that the iron status of some children deteriorated during treatment, particularly with the WHO recommended lower iron formula, indicating that the recommended level of iron in RUTF is less effective in covering the increased need for iron during rapid catch up [91]. This finding is in line with that of an earlier study conducted in Senegal that showed that the currently recommended iron density in RUTFs is not sufficient to cover the needs of children with SAM during the period of rapid catch up growth, particularly in those children with higher weight gain velocities [37].

This study also provided evidence that current anthropometric discharge criteria are not a good proxy for attainment of homeostasis for iron, with anthropometric discharge criteria being met before a significant proportion of children attains replenished BIS, especially those who were depleted at the start of treatment. Similar issues have been shown in a recent study on immunity in children recovering from SAM [92], and taken together indicate the need for further research to determine the optimum criteria of discharging children that takes into account the fact that RUTF intake should not be prolonged unnecessarily given its high content of fat and other nutrients, and the need to minimize risk of relapse due to excessive morbidity. In parallel to the determination of new discharge criteria, there is a need to accelerate research aiming at determining which follow up intervention may ensure post-discharge recovery of iron homeostasis. Several studies have recently published results of interventions that can be combined with CMAM to ensure such post-discharge recovery [72, 93, 94]. With the increasing interest in ensuring long term benefits of SAM treatment, such interventions may contribute to the reduction of excessive morbidity observed during the first 3 months after discharge. Indeed, the high prevalence of ID and IDA among the breastfed children with SAM in this study points toward insufficient iron intake from the readily available complementary foods used by children below 5 years of age in communities where children with SAM come from [95, 96].

This study demonstrates that RUTFs should always have an appropriate density of bioavailable iron to enable reversal of ID and IDA. The FSMS-RUTF iron profile can serve as a starting point for further research and improvements that take into account the effect of cow’s milk and other ingredients and factors such as phytate in the food. As an important proportion of children recovering from SAM meet anthropometric criteria for cure before reversal of their IDA, there is a need to develop an appropriate post-discharge follow up package for continuation of ID correction such as proper administration of well-formulated micronutrient powders or lipid-based nutrients supplements, anti-helminth and anti-malaria treatments, and improvements of environmental hygiene.

Our results have to be interpreted taking into account some methodological limitations. The first is that we used indicators of iron status that are influenced by inflammation and a high proportion of children enrolled into the study had inflammation at admission. To address this, we adjusted these parameters for inflammation [58, 97] and used the sTfr and the sTfr/ferritin index to define the iron status [98]. Studies have shown that these are less affected by inflammation and are good indicators of iron status in Malawian children [42, 56, 58, 99] and we therefore believe that our estimates are the most precise that can practically be obtained in the absence of bone marrow or liver biopsy analysis. Although serum ferritin was also measured, we did not use this biomarker for defining iron status in this study because of the clear absence of correlation of this biomarker and anaemia in our study population. This finding was in accordance with findings from other teams showing that serum ferritin is less accurate in assessing ID in Malawian children even after adjustment for inflammation as malaria infection may lead to long lasting ferritin elevation [42, 100]. The second limitation is the absence of screening for malaria and any inherited disorders known to influence the markers of iron status or the biological regulation of iron homeostasis [16, 101–103]. This may have resulted in overestimating the prevalence of ID and IDA. However, as this study was embedded in an RCT with good randomisation at baseline, it is unlikely that this factor introduced a difference between the arms. Finally, because of financial constraints we were not able to include any analysis of the microbiota. This could have permitted a better understanding of the bacterial population of the gut at the onset and during the course of treatment and enabled us to ascertain the safety of iron densities used with regard to pathogenic gut bacterial growth and selection. Moreover, having data on microbiota changes could have allowed us to examine the influence of the microbiota on the observed difference in treatment effect on haemoglobin and iron status.

## Conclusion

Our study shows that anaemia including IDA is common among children with SAM and this justifies presumptive treatment against ID and IDA in this group. The study also indicates that the FSMS-RUTF with a higher level of iron and no cow’s milk is more efficacious in correcting anaemia and BIS among children with SAM than the current standard milk-based formulation and that raising the levels of iron in RUTF was not associated with any adverse outcomes.

## Additional files

Additional file 1: Predictors of anaemia at admission. (DOCX 13 kb)

Additional file 2: Predictors of iron deficiency anaemia at admission. (DOCX 14 kb)

Additional file 3: Effect of treatment on haemoglobin levels in anaemic and non-anaemic children with SAM across study arms. (DOCX 14 kb)

Additional file 4: Predictors of anaemia at discharge. (DOCX 13 kb)

Additional file 5: Change in body iron stores (BIS) of children with SAM across study arms. (DOCX 15 kb)

## Data Availability

The datasets used and/or analysed during the current study are available from the corresponding author on reasonable request.

## Abbreviations

AGP: Alpha-1-acid glycoprotein
BIS: Body iron stores
CI: Confidence interval
CMAM: Community-based management of acute malnutrition
FSMS-RUTF: Milk-free Soybean-Maize-Sorghum based ready-to-use therapeutic food
ID: Iron deficiency
IDA: Iron deficiency anaemia
IQR: Interquartile range
ITN: Insecticide treated net
LNS: Lipid-based nutrient supplement
MCV: Mean copuscular volume
MSMS-RUTF: Milk soybean-maize-sorghum based ready-to-use therapeutic food
MUAC: Mid-upper arm circumference
OR: Odds ratio
PM-RUTF: Peanut and milk ready-to-use therapeutic food
RBC: Red blood cell count
RE: Retinol equivalents
RR: Relative risk
RUTF: Ready-to-use therapeutic food
SAM: Severe acute malnutriton
SD: Standard deviation
SMS-RUTF: Soybean-maize-sorghum based ready-to-use therapeutic food
sTfR: Soluble transferrin receptor
TE: Tocopherol equivalents
WHO: World Health Organization
