# Supplementary material for: Soya, maize and sorghum ready-to-use therapeutic foods are more effective in correcting anaemia and iron deficiency than the standard ready-to-use therapeutic food: randomized controlled trial
Source: BMC Public Health. 2019 Jun 24;19:806. doi: 10.1186/s12889-019-7170-x (PMC6591918; doi:10.1186/s12889-019-7170-x)
Supplement: Supplementary file 1 — Predictors of anaemia at admission. (DOCX 13 kb) [file 12889_2019_7170_MOESM1_ESM.docx]

Additional file 1: Predictors of anaemia at admission (n=346)

| Parameters | AOR | (95%CI) | p-value |
| --- | --- | --- | --- |
| Inflammation category |  |  |  |
| Incubation | 5.1 | (1.6; 16.8) | 0.007 |
| Early convalescence | 6.5 | (2.6; 16.4) | <0.001 |
| Late convalescence | 1.5 | (0.7; 3.2) | 0.304 |
| No inflammation | 1.0 |  |  |
| Sex (Female/male) | 1.6 | (0.9; 2.7) | 0.097 |
| Age at admission (months) | 0.9 | (0.9; 1.0) | 0.001 |
| β-thalassemia trait(Present /absent) | 0.4 | (0.2; 0.9) | 0.036 |
| Weight at admission (kg) | 1.5 | (1.1; 2.0) | 0.016 |
| Constant | 1.7 | (0.1; 46.6) |  |

Not retained in the model: breastfeeding status, MUAC at admission and oedema
