# Supplementary material for: Soya, maize and sorghum ready-to-use therapeutic foods are more effective in correcting anaemia and iron deficiency than the standard ready-to-use therapeutic food: randomized controlled trial
Source: BMC Public Health. 2019 Jun 24;19:806. doi: 10.1186/s12889-019-7170-x (PMC6591918; doi:10.1186/s12889-019-7170-x)
Supplement: Supplementary file 2 — Predictors of iron deficiency anaemia at admission. (DOCX 14 kb) [file 12889_2019_7170_MOESM2_ESM.docx]

Additional file 2: Predictors of iron deficiency anaemia at admission (n=335)

| Parameters | OR | (95%CI) | p-value |
| --- | --- | --- | --- |
| Inflammation category |  |  |  |
| Incubation | 5.3 | (1.3; 20.9) | 0.007 |
| Early convalescence | 4.7 | (1.9; 11.6) | <0.001 |
| Late convalescence | 2.0 | (0.7; 5.7) | 0.174 |
| No inflammation | 1.0 |  |  |
| Breastfeeding (Yes/not) | 2.3 | (1.2; 4.6) | 0.016 |
| Age at admission (months) | 0.9 | (0.9; 1.0) | 0.004 |
| β-thalassemia trait(Present /absent) | 0.4 | (0.2; 1.0) | 0.052 |
| Weight at admission (kg) | 1.6 | (1.1; 2.2) | 0.023 |
| MUAC at admission (mm) | 1.0 | (0.9; 1.0) | 0.089 |
| Constant | 1.4 | (0.1; 24.1) |  |

Not retained in the model: sex and oedema
