# Supplementary material for: Soya, maize and sorghum ready-to-use therapeutic foods are more effective in correcting anaemia and iron deficiency than the standard ready-to-use therapeutic food: randomized controlled trial
Source: BMC Public Health. 2019 Jun 24;19:806. doi: 10.1186/s12889-019-7170-x (PMC6591918; doi:10.1186/s12889-019-7170-x)
Supplement: Supplementary file 3 — Effect of treatment on haemoglobin levels in anaemic and non-anaemic children with SAM across study arms. (DOCX 14 kb) [file 12889_2019_7170_MOESM3_ESM.docx]

Additional file 3: Effect of treatment on haemoglobin levels in anaemic and non-anaemic SAM children across study arms

|  | **Category** | ***n*** | **Admission Hb**  **mean (SD)** | **N** | **Discharge Hb**  **mean (SD)** | **Difference,**  **Δ(95%CI)** | **p-value** |
| --- | --- | --- | --- | --- | --- | --- | --- |
| All | FSMS-RUTF | 134 | 11.0 (2.0) | 83 | 12.1 (1.2) | 1.1(0.6; 1.6) | <0.001 |
|  | MSMS-RUTF | 109 | 10.5 (2.1) | 77 | 11.8 (1.2) | 1.3 (0.7; 1.8) | <0.001 |
|  | PM-RUTF | 149 | 10.7 (1.79) | 106 | 11.6 (1.2) | 0.9 (0.5; 1.4) | <0.001 |
|  | p-value |  | 0.879 |  | 0.132 |  |  |
| Anaemic | FSMS-RUTF | 58 | 9.1 (1.3) | 38 | 12.1 (1.1) | 2.9 (2.4; 3.4) | <0.001 |
|  | MSMS-RUTF | 59 | 9.1 (1.4) | 40 | 11.4 (1.5) | 2.3 (1.8; 2.9) | <0.001 |
|  | PM-RUTF | 72 | 9.0 (1.4) | 45 | 11.2 (1.2) | 2.2 (1.7; 2.7) | <0.001 |
|  | p-value |  | 0.863 |  | 0.007 |  |  |
| Non-anaemic | FSMS-RUTF | 72 | 12.5 (1.1) | 43 | 12.2 (1.2) | -0.2 (-0.7; 0.2) | 0.290 |
|  | MSMS-RUTF | 48 | 12.3 (0.9) | 35 | 11.2 (0.8) | -0.1(-0.5; 0.3) | 0.553 |
|  | PM-RUTF | 77 | 12.2 (0.7) | 59 | 11.9 (1.1) | -0.3 (-0.6; 0.0) | 0.074 |

Hb, haemoglobin (g/L); Study arms: FSMS-RUTF= Milk Free Soya-Maize-Sorghum Based Ready-To-Use Therapeutic Food, MSMS-RUTF=Milk Soya-Maize-Sorghum Based Ready-To-Use Therapeutic Food and PM-RUTF= Peanut milk based Ready-To-Use Therapeutic Food.
