# Supplementary material for: Soya, maize and sorghum ready-to-use therapeutic foods are more effective in correcting anaemia and iron deficiency than the standard ready-to-use therapeutic food: randomized controlled trial
Source: BMC Public Health. 2019 Jun 24;19:806. doi: 10.1186/s12889-019-7170-x (PMC6591918; doi:10.1186/s12889-019-7170-x)
Supplement: Supplementary file 4 — Predictors of anaemia at discharge. (DOCX 13 kb) [file 12889_2019_7170_MOESM4_ESM.docx]

Additional file 4: Predictors of anaemia at discharge (n=262)

| Parameters | OR | (95%CI) | p-value |
| --- | --- | --- | --- |
| Study arm |  |  |  |
| FSMS-RUTF | 0.3 | (0.2; 0.5) | <0.001 |
| MSMS-RUTF | 0.6 | (0.3; 1.0) | 0.068 |
| PM-RUTF | 1.0 |  |  |
| Haemoglobin at admission (g/dL) | 0.6 | (0.5; 0.8) | <0.001 |
| RUTF intake (sachets/day) | 0.6 | (0.5; 0.8) | <0.001 |
| Constant | 130.6 | (22.0; 775.2) |  |

Not retained in the model: sex and oedema at admission, age at admission, breastfeeding status at admission and length of stay in programme, fever, diarrhea, cough, abdominal pain
