# Supplementary material for: Soya, maize and sorghum ready-to-use therapeutic foods are more effective in correcting anaemia and iron deficiency than the standard ready-to-use therapeutic food: randomized controlled trial
Source: BMC Public Health. 2019 Jun 24;19:806. doi: 10.1186/s12889-019-7170-x (PMC6591918; doi:10.1186/s12889-019-7170-x)
Supplement: Supplementary file 5 — Change in body iron stores (BIS) of children with SAM across study arms. (DOCX 15 kb) [file 12889_2019_7170_MOESM5_ESM.docx]

Additional file 5: Change in body iron stores (BIS) of SAM children across study arms

|  | **Study arm^1^** | ***n*** | **Admission BIS,**  **mean (SD)** | **n** | **Discharge BIS,**  **mean (SD)** | **Difference,**  **Δ (95%CI)** | **p-value^2^** |
| --- | --- | --- | --- | --- | --- | --- | --- |
| All | FSMS-RUTF | 115 | 1.9 (4.3) | 64 | 3.9 (2.7) | 2.0 (1.0;3.1) | <0.001 |
|  | MSMS-RUTF | 92 | 2.0 (4.1) | 46 | 3.1 (3.3) | 1.1 (-0.2;2.4) | 0.093 |
|  | PM-RUTF | 136 | 2.0 (4.3) | 84 | 2.0 (3.4) | 0.0 (-1.0;1.1) | 0.962 |
|  | p-value^3^ |  | 0.972 |  | 0.001 | 0.011 |  |
| BIS≥3 mg/kg | FSMS-RUTF | 48 | 5.4 (1.8) | 22 | 4.5 (1.9) | -0.9 (-1.9;0.1) | 0.074 |
|  | MSMS-RUTF | 44 | 5.2 (1.6) | 19 | 4.9 (3.2) | -0.3 (-1.9;1.3) | 0.686 |
|  | PM-RUTF | 61 | 5.7 (1.8) | 35 | 3.5 (2.9) | -2.2 (-3.3; -1.1) | <0.001 |
|  | p-value |  | 0.335 |  | 0.173 | 0.112 |  |
| 0<BIS<3 mg/kg | FSMS-RUTF | 39 | 1.7 (0.9) | 21 | 3.9 (2.8) | 2.2 (0.8; 3.5) | 0.003 |
|  | MSMS-RUTF | 27 | 1.3 (0.9) | 12 | 3.2 (1.4) | 1.9 (0.9; 2.8) | <0.001 |
|  | PM-RUTF | 34 | 1.4 (1.0) | 22 | 2.5 (2.2) | 1.1 (0.1; 2.1) | 0.033 |
|  | p-value |  | 0.118 |  | 0.175 | 0.224 |  |
| BIS≤0 mg/kg | FSMS-RUTF | 28 | -3.9 (3.6) | 13 | 2.2 (3.4) | 6.2 (3.7; 8.6) | <0.001 |
|  | MSMS-RUTF | 21 | -3.8 (3.5) | 8 | -0.6 (2.4) | 3.2 (0.8; 5.6) | 0.011 |
|  | PM-RUTF | 41 | -3.2 (2.9) | 20 | -0.9 (4.0) | 2.2 (0.2; 4.3) | 0.034 |
|  | p-value |  | 0.551 |  | 0.0478 | 0.045 |  |

^1^Study arms: FSMS-RUTF= Milk Free Soya-Maize-Sorghum Based Ready-To-Use Therapeutic Food, MSMS-RUTF=Milk Soya-Maize-Sorghum Based Ready-To-Use Therapeutic Food and PM-RUTF= Peanut milk based Ready-To-Use Therapeutic Food;  ^2^ p-value for t-test ; ^3^p-value for ANOVA analysis
